# The impact of incident stroke on cognitive trajectories in later life

**DOI:** 10.1186/s13195-024-01479-8

**Published:** 2024-05-18

**Authors:** Swarna Vishwanath, Ingrid Hopper, Geoffrey C. Cloud, Trevor T-J Chong, Raj C. Shah, Geoffrey A. Donnan, Jeff D. Williamson, Charles B. Eaton, Rory Wolfe, Christopher M. Reid, Andrew M. Tonkin, Suzanne G. Orchard, Sharyn Fitzgerald, Anne M. Murray, Robyn L. Woods, Mark R. Nelson, Ajay Sood, Claire J. Steves, Joanne Ryan

**Affiliations:** 1 School of Public Health and Preventive Medicine, Monash University, 553 St Kilda Road, Melbourne, VIC 3004 Australia; 2 Department of Neuroscience, Central Clinical School, Monash University, Melbourne, Australia; 3 Department of Cardiology and General Medicine Unit, Alfred Health, Melbourne, Australia; 4 Department of Neurology, Alfred Health, Melbourne, VIC Australia; 5 Turner Institute for Brain and Mental Health, School of Psychological Sciences, Monash University, Clayton, VIC Australia; 6 Department of Clinical Neurosciences, St. Vincent’s Hospital, Melbourne, VIC Australia; 7 Department of Family and Preventive Medicine, Rush Alzheimer’s Disease Center, Rush University Medical Center, Chicago, IL USA; 8 Melbourne Brain Centre, University of Melbourne, Royal Melbourne Hospital, Melbourne, Australia; 9 Sticht Center for Healthy Aging and Alzheimer’s Prevention, Department of Internal Medicine, Section on Geriatric Medicine, Wake Forest School of Medicine, Winston-Salem, NC USA; 10 Department of Family Medicine and Epidemiology, Brown University Warren Alpert Medical School and School of Public Health, Pawtucket, RI USA; 11 School of Population Health, Curtin University, Western Australia, Australia; 12 Berman Center for Outcomes & Clinical Research, Hennepin Healthcare Research Institute, Minneapolis, MN USA; 13 Department of Medicine, Division of Geriatrics Hennepin Healthcare, University of Minnesota, Minneapolis, MN USA; 14 Menzies Institute for Medical Research, University of Tasmania, Hobart, TAS Australia; 15 Department of Twin Research & Genetic Epidemiology, King’s College London, London, UK

**Keywords:** Cognitive function, Cognitive domains, Stroke, Trajectories, Dementia, Linear mixed model

## Abstract

**Background:**

Cognitive impairment is common after stroke, and a large proportion of stroke patients will develop dementia. However, there have been few large prospective studies which have assessed cognition both prior to and after stroke. This study aims to determine the extent to which incident stroke impacts different domains of cognitive function in a longitudinal cohort of older community-dwelling individuals.

**Methods:**

19,114 older individuals without cardiovascular disease or major cognitive impairment were recruited and followed over a maximum 11 years. Stroke included ischaemic and haemorrhagic stroke and was adjudicated by experts. Cognitive function was assessed regularly using Modified Mini-Mental State Examination (3MS), Hopkins Verbal Learning Test–Revised (HVLT-R), Symbol Digit Modalities Test (SDMT), and Controlled Oral Word Association Test (COWAT). Linear mixed models were used to investigate the change in cognition at the time of stroke and decline in cognitive trajectories following incident stroke.

**Results:**

During a median follow-up period of 8.4 [IQR: 7.2, 9.6] years, 815 (4.3%) participants experienced a stroke. Over this time, there was a general decline observed in 3MS, HVLT-R delayed recall, and SDMT scores across participants. However, for individuals who experienced a stroke, there was a significantly greater decline across all cognitive domains immediately after the event immediately after the event (3MS: -1.03 [95%CI: -1.45, -0.60]; HVLT-R: -0.47 [-0.70, -0.24]; SDMT: -2.82 [-3.57, -2.08]; COWAT: -0.67 [-1.04, -0.29]) and a steeper long-term decline for three of these domains (3MS -0.62 [-0.88, -0.35]; COWAT: -0.30 [-0.46, -0.14]); HVLT-R: -0.12 [95%CI, -0.70, -0.24]). However individuals with stroke experienced no longer-term decline in SDMT compared to the rest of the participants.

**Conclusions:**

These findings highlight the need for comprehensive neuropsychology assessments for ongoing monitoring of cognition following incident stroke; and potential early intervention.

**Supplementary Information:**

The online version contains supplementary material available at 10.1186/s13195-024-01479-8.

## Background

Stroke ranks among the leading causes of adult disability globally [1] with notable increases in the overall stroke burden especially among the older adult population [2]. Additionally, advances in treatment for acute stroke have led to a decline in fatality rates, resulting in an increase in the number of stroke survivors [3]. Among the many challenges facing stroke survivors [3] is the risk of cognitive decline and dementia, which become increasingly common after the age of 70 years [4].

Stroke doubles the risk of dementia, with improved survival following stroke, the prevalence of dementia after stroke is increasing [5]. Impaired cognitive function is also common among stroke survivors though it is important to note that cognitive decline after incident stroke may be confounded by existing cognitive decline or dementia prior to the stroke [6]. To accurately assess the impact of stroke on cognition, it is important to include cognitive assessments prior to occurrence of stroke. However, most studies conducted thus far have only assessed cognition after incident stroke [7]. There are few studies that have data on individual cognitive trajectories that describe the course of cognitive change over time [8]. A large study measuring cognitive changes after controlling for pre-stroke cognition in individuals aged 45 years and older reported both acute and long-term declines in cognition after stroke, however, the mean ages of participants in this study was below 65 years [9]. Similar studies have not been performed in older adults, and it is unclear whether the impact of stroke on cognitive function in older adults demonstrates a similar pattern, or has an even greater impact on the ageing brain.

Furthermore, there are different domains of cognitive function and stroke may affect these domains differently. Identifying domain-specific changes in cognitive function after stroke would help inform cognitive rehabilitation programs which will optimize cognitive recovery following stroke [10]. Hence, the aim of our study was to determine whether incident stroke impacts cognitive aging trajectories in a large prospective cohort of community-dwelling older individuals with no prior cardiovascular disease events or major cognitive impairment. The secondary aim of this study was to examine the incidence rates of dementia after stroke and to compare the rates with those who did not have stroke.

## Methods

### Study population

Community-dwelling adults from Australia and the United States (US) aged 70 years and older (65 for African-American and Hispanic communities in the US) and were recruited to the ASPirin in Reducing Events in the Elderly (ASPREE) randomized clinical trial (RCT) between 2010 and 2014 and followed prospectively until June 2017 [11]. Details of inclusion/exclusion criteria for ASPREE participants have been published elsewhere [11]. Highly relevant to this study, eligible participants did not have diagnosed cardiovascular disease (defined as myocardial infarction, heart failure, angina pectoris, stroke, transient ischemic attack, > 50% carotid stenosis or previous carotid endarterectomy or stenting, coronary artery angioplasty or stenting, coronary artery bypass grafting, abdominal aortic aneurysm) or a clinical diagnosis of dementia or major cognitive impairment (a score of < 78 out of 100 on Modified Mini-Mental State (3MS) examination at baseline). ASPREE was a double-blind, placebo-controlled trial investigating the effect of low-dose aspirin on disability-free survival in healthy older adults. Stroke and dementia were prespecified endpoints for ASPREE. The main trial results reported no significant effect of aspirin on either of these outcomes [12–14]. Ongoing observational follow-up of participants annually occurs through the ASPREE eXTension (ASPREE-XT) study [15]. Further details on the study design and health characteristics for ASPREE and ASPREE-XT are published [15, 16].

### Stroke

Stroke was a prespecified secondary endpoint and events were adjudicated by an expert panel [12, 13]. This included: (i) fatal stroke, defined as any death in which the underlying cause was an obstruction or rupture in the intracranial or extracranial cerebral arterial system; and (ii) non-fatal stroke, defined based on the World Health Organization definition of rapidly developing clinical signs of focal or global disturbance of cerebral function lasting more than 24 h (unless interrupted by surgery or death), with no apparent cause other than ischaemic or haemorrhagic cerebrovascular disease [17].

### Cognitive assessments

Cognitive assessments were administered three times every other year over the first 5 years, and annually thereafter by trained staff over a maximum of eleven years. The cognitive battery included the Modified Mini-Mental State Examination (3MS), a measure of global cognition, the Hopkins Verbal Learning Test-Revised delayed recall (HVLT-R) task for episodic memory, the Symbol Digit Modalities Test (SDMT) to measure processing speed, and the single letter (F) Controlled Oral Word Association Test (COWAT) for executive function and verbal fluency.

### Dementia

Participants with suspected dementia (3MS score ≤ 77, decline of > 10.15 points from the predicted 5-year score, reported cognitive concerns in medical records, a clinician diagnosis of dementia, or prescription of cholinesterase inhibitors and other dementia medications) were referred for further cognitive and functional testing including Alzheimer’s Disease Assessment Scale – cognitive subscale, [18] Colour trails, [19] Lurian overlapping figures, [20] and the Alzheimer Disease Cooperative Study Activities of Daily Living Scale [21]. A specialist committee composed of geriatricians and neurologists reviewed this information together with detailed medical history and the results of clinical exams and bloods, where available [12]. Dementia was adjudicated according to the criteria found in the Diagnostic and Statistical Manual of Mental Disorders, Fourth Edition [22].

### Covariates

Baseline demographic factors considered were age, sex, years of education (< 12 years or ≥ 12 years), country/ethno-racial group (Australian white, American white, black/African-American, or Hispanic/Latino/Other). Health-related behaviours considered were baseline smoking status (never, former, or current) and baseline alcohol consumption status (never, former, or current). Clinical factors considered were hypertension (yes or no, based on whether the mean of three blood pressure measurements was systolic blood pressure ≥ 140 mmHg or diastolic blood pressure ≥ 90 mmHg and/or whether the participants had prescription for anti-hypertensive medication), diabetes mellitus (yes or no, based on self-report or fasting glucose ≥ 126 mg/dL [≥ 7 mmol/L] or on treatment for diabetes), obesity (BMI ≥ 30 kg/m^2^), dyslipidaemia (yes or no, based on the use of cholesterol-lowering medications or low-density lipoprotein, LDL > 160 mg/dL [> 4.1mmol/L] or serum cholesterol ≥ 212 mg/dL [≥ 5.5 mmol/L] for Australians or ≥ 240 mg/dL [≥ 6.2 mmol/L] for US participants), and depressive symptoms (score of ≥ 8/30, measured by the Centre for Epidemiologic Studies Depression scale [CESD] 10).

### Statistical analyses

The statistical analysis was performed in Stata (version 17.0, Stata Corp, College Station, TX). Descriptive characteristics were presented according to incident stroke. Incidence rates for dementia were also calculated following incident stroke and for those without stroke. Linear mixed models were used to estimate cognitive change over time, separately for the 4 cognitive tests. Significance level was set at *p* < 0.05 and the tests were 2-sided. Due to the small number of participants with cognitive assessments at year 2 (Supplementary figure SF1), the scores from this timepoint was not included in the analysis.

The independent variables included in each model were (1) the study follow-up time, expressed in years from the date of recruitment; (2) a time-varying binary variable, which changed from 0 to 1 after incident stroke; and (3) another time variable representing the time after stroke, expressed in years from the date of incident stroke. The effect size associated with the study follow-up time variable represents the annual change in cognition scores for all participants over the follow-up period while they remain stroke free. The effect size of the time-varying variable represents the acute decline in cognition after incident stroke, [9] which was defined as the change in cognition in the time immediately after stroke and was estimated based on the first cognitive assessment administered after incident stroke as well as other cognition scores before and after stroke [9]. Lastly, the effect size for the time after stroke variable represents the annual change in cognition scores after incident stroke. The models included both random intercept and slope effects to account for participant specific individual differences. An unstructured correlation matrix was specified and the model was fitted using maximum likelihood. Sensitivity analyses were conducted for the cognitive trajectory analysis by: (1) excluding participants who died during the study period [since death is a competing risk factor]; (2) excluding participants who were adjudicated with dementia through the study period; (3) aetiological sub-groups of strokes, .i.e. ischaemic and haemorrhagic.

## Results

Among the 19,114 ASPREE participants, there were 815 (4.3%) cases of incident stroke during a median [IQR] follow-up of 8.4 [7.2, 9.6] years. Table 1 includes the descriptive statistics by incident stroke status for all participants. The mean cognitive scores for the sample population over the 11 years of follow-up is included in supplementary table ST1. Individuals who experienced incident stroke had a median age of 76.0 [73.0, 80.7], a total of 48.6% of those were males. Among those who experienced an incident stroke, at the initial (baseline) assessment, 13.5% had diabetes, 78.9% hypertension, and 12.1% with depressive symptoms (Table 1). The median[IQR] time from the stroke event to the first cognitive assessment was 0.62 [0.31, 1.04] years (mean (SD) – 0.76 (0.64)) and the median [IQR] follow-up time after incident stroke was 2.82 [1.03, 5.38] years.

**Table 1:** Descriptive characteristics at baseline of the sample population by stroke status (*n* = 19,114)

	All	Stroke	No Stroke
n	19,114 (100%)	815 (4.3%)	18,299 (95.7%)
Age (y) Median [IQR]	74 [71.6–77.7]	76.0 [73.0-80.7]	73.9 [71.6–77.5]
Male	8,332 (43.6%)	396 (48.6%)	7,936 (43.4%)
Education (y)			
< 12	8,636 (45.2%)	391 (48.0%)	8,245 (45.1%)
≥ 12	10,477 (54.8%)	424 (52.0%)	10,053 (54.9%)
Ethno-racial group			
White Australian	16,361 (85.6%)	707 (86.7%)	15,654 (85.5%)
White American	1,088 (5.7%)	55 (6.7%)	1,033 (5.6%)
Black / African-American	905 (4.7%)	25 (3.1%)	880 (4.8%)
Other^	750 (3.9)	28 (3.4%)	732 (4.0%)
Smoking status			
Current	735 (3.8%)	45 (5.5%)	690 (3.8%)
Former	7,799 (40.8%)	342 (42.0%)	7,457 (40.8%)
Never	10,580 (55.4%)	428 (52.5%)	10,152 (55.5%)
Alcohol consumption			
Current	14,642 (76.6%)	628 (77.1%)	14,014 (76.6%)
Former	1,136 (5.9%)	42 (5.2%)	1,094 (6.0%)
Never	3,336 (17.5%)	145 (17.8%)	3,191 (17.4%)
Diabetes	2,045 (10.7%)	110 (13.5%)	1,935 (10.6%)
Hypertension	14,195 (74.3%)	643 (78.9%)	13,553 (74.1%)
Depression symptoms*	1,879 (9.8%)	99 (12.1%)	1,780 (9.7%)
3MS, mean (SD)	93.4 (4.6)	93.0 (4.6)	93.4 (4.6)
HVLT-R, mean(SD)	7.7 (2.8)	7.4 (2.9)	7.7 (2.8)
COWAT, mean (SD)	12.1 (4.6)	11.8 (4.6)	12.1 (4.6)
SDMT, mean (SD)	36.7 (10.1)	34.4 (10.3)	36.8 (10.1)

Notes:

*Abbreviations*: n – sample size; y – years; IQR – interquartile range; SD – standard deviation; 3MS – modified mini-mental state exam; HVLT-R – Hopkins verbal learning test-revised delayed recall; COWAT – single letter controlled oral word association test, Letter F; SDMT – symbol digit modalities test

^ Hispanic or Latino / Asian / Other

* *Depression symptoms* defined as CESD-10 score of 8+/30.

* *Sample size (where different from overall sample size of n = 19,114)*

Education *n* = 19,113; HVLT-R *n* = 19,007; COWAT *n* = 19,083; SDMT *n* = 19,030

### Overall change in cognition

**Fig. 1:**
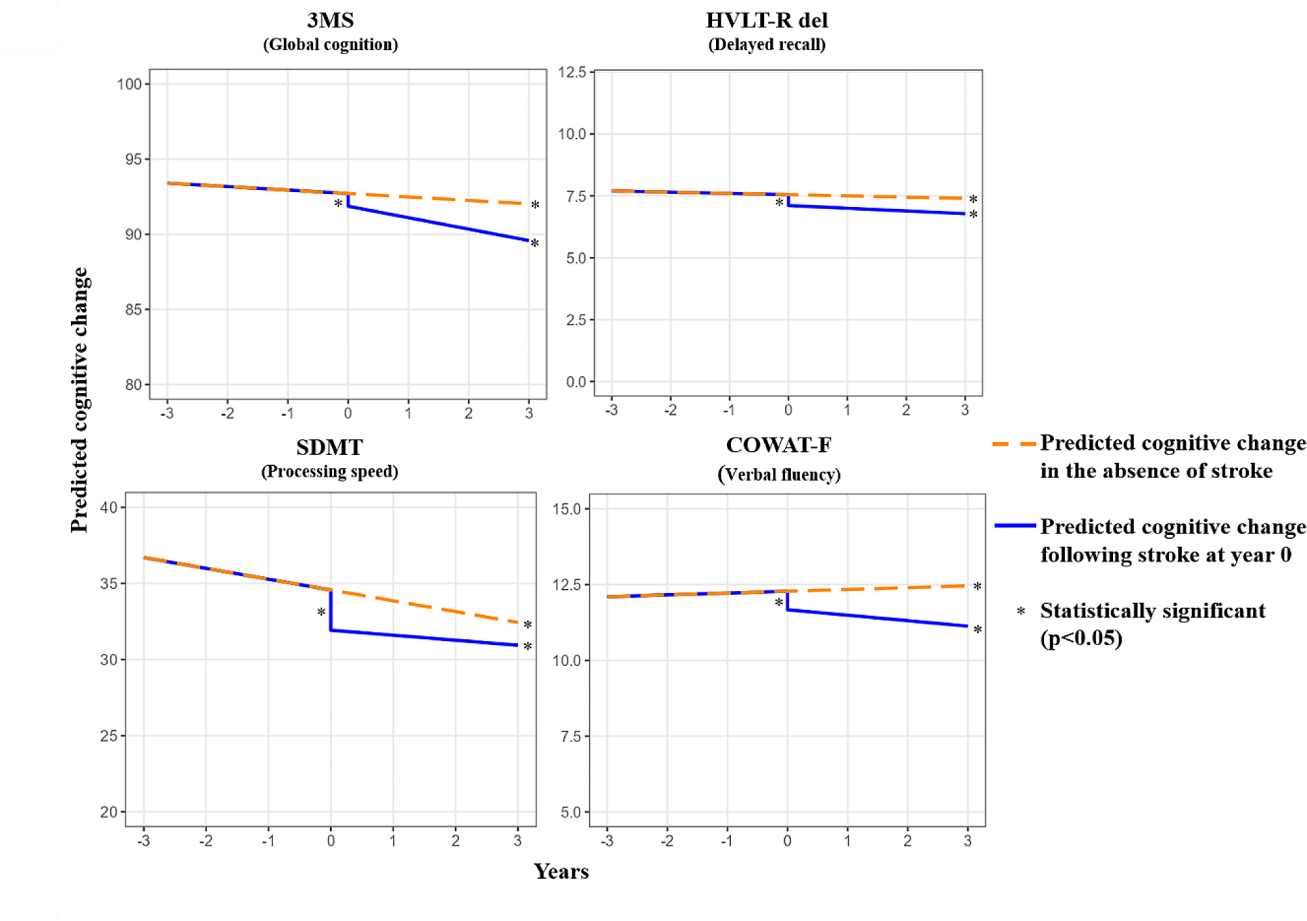
Predicted change in cognitive function after stroke (centred at time 0) Note: Results from mixed linear regression models with participant-specific random intercept and slope adjusting for overall follow-up time (in years), a time-varying stroke variable (centred at year 0, .i.e. stroke occurred at year 0), time after an incident stroke (includes time points after 0). Other covariates included in the models were sex, age, ethnicity and country, education, smoking, alcohol consumption, depressive symptoms, diabetes, and baseline cognition score. Abbreviations: 3MS, Modified Mini Mental State Exam; HVLT-R, Hopkins Verbal Learning Test-Revised Delayed; SDMT, Symbol Digit Modalities Test; COWAT – single letter controlled oral word association test, Letter F

The overall (*n* = 19,114) participant mean (SD) score on 3MS at baseline was 93.4 (4.6) (Table 1) and estimated annual 3MS decline was − 0.20 [-0.21, -0.18] (Table 2; Fig. 1). The mean (SD) HVLT-R score at baseline was 7.7(2.8) (Table 1) which declined annually by -0.04[-0.04, -0.03] over study follow-up (Table 2; Fig. 1). For SDMT, the mean(SD) baseline scores when considering all participants was 36.7(10.1) (Table 1) and there was an annual change of -0.68[-0.70,-0.67] (Table 2; Fig. 1). Finally, for COWAT the baseline mean (SD) scores for all participants was 12.1(4.6) and there was an annual increase in verbal fluency (0.09 [0.08 to 0.10]) (Fig. 1; Table 2) across the study follow-up period.

**Table 2:** Stroke and change in cognitive performance over time (*n* = 19,114)

	3MS (Global cognition)	HVLT-*R* (Episodic memory)	SDMT (Processing speed)	COWAT -F (Verbal fluency)
Overall cognitive change over time *	**-0.20** **[-0.21, -0.18]**	**-0.04** **[-0.04, -0.03]**	**-0.68** **[-0.70, -0.67]**	**0.09** **[0.08, 0.10]**
Acute cognitive change after stroke^#^	**-1.03** **[-1.45, -0.60]**	**-0.47** **[-0.70, -0.24]**	**-2.82** **[-3.57, -2.08]**	**-0.67** **[-1.04, -0.29]**
Cognitive change over time after stroke^	**-0.75** **[-0.98, -0.53]**	**-0.12** **[-0.70, -0.24]**	**-0.30** **[-0.56, -0.05]**	**-0.17** **[-0.28, -0.07]**

Note: This table includes mixed linear regression results with participant-specific random intercept and slope adjusting for age, sex, country and ethnicity, education, smoking, alcohol, diabetes, depressive symptoms and including time (years) and a time-varying stroke variable (all participants were event-free at baseline), and a time variable representing the time (years) after a stroke. Potential confounding factors not significant in the univariate analyses (hypertension, obesity, dyslipidaemia, and chronic kidney disease) were not included as covariates

Bold represents s**ignificant** result *p* < 0.05

* This effect size shows the annual change in cognitive scores on each of the four tests for all participants

^#^ This effect size shows the acute change in cognitive scores following a stroke (at the first cognitive assessment after stroke)

^ This effect size shows the annual change in cognitive scores on each of the four tests after stroke

*Abbreviations*: n – sample size; 3MS – Modified Mini-Mental State exam; HVLT-R – Hopkins verbal learning test-revised; SDMT – symbol digit modalities test; COWAT – single letter controlled oral word association test, Letter F

### Impact of incident stroke on Cognition

At the first cognitive assessment following incident stroke, there was a significant decrease in all four tests (3MS: -1.03 [-1.46, -0.60]; HVLT-R: -0.47 [-0.70, -0.24]; SDMT: -2.82 [-3.57, -2.08]; and COWAT: -0.67 [-1.04, -0.29]) (Fig. 1; Table 2). In the years following incident stroke, there was annual decline in 3MS (-0.75 [-0.98, -0.53]), HVLT-R (-0.12 [-0.21, -0.03]), SDMT (-0.30 [-0.56, -0.05]) and COWAT (-0.17 [-0.28, -0.07]) (Fig. 1; Table 2). Supplementary table ST2 includes results for all covariates included in the linear mixed models.

### Dementia after stroke

Out of the 815 participants with stroke during this study, a total of 76 (9.3%) individuals were adjudicated as having incident dementia as well. Thirty-one (40.8%) participants had dementia confirmed after incident stroke, and the remaining 45 (59.2%) participants were confirmed as having dementia prior to stroke. In the non-stroke cohort (*n* = 18,299) there were a total of 1,141 (6.2%) participants adjudicated as having incident dementia.

### Sensitivity analyses

After excluding 3028 participants who died during the study period, the results were comparable (supplementary table ST3; *n* = 16,086) to the main analyses. Similarly, when excluding participants with dementia (*n* = 1217), the results followed the same pattern as the main findings, however, the effect sizes had reduced and confidence intervals were wider (supplementary table ST4; *n* = 17,897). For the sub-types of stroke, after a haemorrhagic stroke (*n* = 135, 16.6% of all strokes) there was significant drop in 3MS (-3.71 [-5.01 to -2.41]), HVLT-R (-1.21 [-1.93 to -0.50]) and SDMT (-4.13 [-6.43 to -1.82]) at the first cognitive assessment after stroke and significant annual decline in 3MS (-1.00 [-1.69, -0.31]) and COWAT (-0.43 [-0.82, -0.05]) scores over the years following a haemorrhagic stroke (supplementary table ST5; *n* = 18,434). After an ischaemic stroke (*n* = 596, 73.1% of all strokes) the results were similar to the main analyses (supplementary table ST6; *n* = 18,895).

## Discussion

In this large prospective cohort of community dwelling individuals, predominantly 70 years and older, without cardiovascular disease or major cognitive impairments at baseline, we investigated the individual cognitive trajectories among older individuals and examined the impact of stroke on these cognitive trajectories. A small but a significant drop was observed in all cognitive scores at the first assessment following stroke, and also significant annual decline following stroke in all cognitive domains. The annual decline in 3MS, HVLT-R and COWAT scores accelerated significantly after stroke (∼ 3 times) compared to the average annual decline observed in individuals prior to and without stroke. However, with SDMT scores, our results suggest that there is a period of recovery following the initial drop after incident stroke. The annual decline in SDMT scores is only half the rate observed in individuals prior to and without stroke. These results remained robust in sensitivity analyses after excluding individuals diagnosed with dementia and individuals who died during the study. For the secondary aim, the overall number of dementia cases after incident stroke was lower than the incidence rate of dementia in the no-stroke group. Due to the low numbers of incident dementia after stroke, further statistical testing was not possible.

Prospective studies among adults < 70 years that have investigated the impact of stroke on cognitive function, have predominantly all investigated global cognition, and their findings align with ours [9, 23–25]. Studies investigating processing speed, report cognitive decline in both the short- and long-term, [26–29] which is different to our results. This difference in results could be due to the inclusion of cognitive scores prior to incident stroke in our study whereas previous studies on processing speed predominantly included cognitive assessments after stroke which makes it challenging to account for cognition levels prior to the stroke. Processing speed, defined as how fast information can be processed, is often linked to higher-order cognitive processes [30]. Thus understanding the impact of stroke on this domain is important as it can inform targeted cognitive rehabilitation programs that may benefit both domain specific and generalized cognition [31]. Finally, although cognitive function declines following stroke, the incidence rates of dementia after a stroke were lower compared to incidence rates among those who did not have stroke, which contrasts findings from prior studies that have demonstrated an increased risk of dementia after stroke [7]. However, the relatively short follow-up period after stroke in our study (median ∼ 3 years), combined with the quite small number of dementia cases in this group, could help explain our findings. Dementia is known to have a long pre-clinical phase and a longer follow-up period may be required before such an effect could be observed.

The main clinical implication of this study is that it demonstrates cognitive decline may be expected after stroke in older adults, with varying impact across different cognitive domains and evolving over time after stroke. Although the short-term decline after a stroke was relatively small compared to the Minimal Detectable Change for these cognitive tests [ranging from 6.60 to 9.95 (3MS), 12.42 to 15.61 (SDMT), 6.34 to 8.34 (COWAT-F), 8.13 to 10.85 (HVLT-R total recall), and 4.00 to 5.62 (HVLT-R delayed recall) [32]], the longer-term trajectories following stroke were also significant, indicating a consistent annual decline over time (which would therefore reach clinical significance). AS a consequence, it highlights the importance of using a comprehensive neuropsychological tool to assess the function of various cognitive domains after stroke. The short- and long-term impact of stroke on cognition underscores the need for routine monitoring of cognitive function, starting as early as possible in the stroke journey. In a clinical context, however, accurately assessing cognition after a stroke is made difficult by numerous factors such as delirium at the acute stages of stroke, or depression in the years following stroke. A framework for assessment of cognition in stroke, which emphasised the need for differing approaches to test at differing points in the stroke pathway, has been proposed, however, there is a need for further research in this area [33].

These results also offer implications for cognitive rehabilitation programs after stroke. It suggests that the need for cognitive rehabilitation following stroke varies in the short- and long-term for the different cognitive domains. For example, long-term cognitive rehabilitation programs might benefit from prioritising other cognitive domains rather than processing speed due to the improvements in processing speed over time after incident stroke. Furthermore, research indicates that the effectiveness of cognitive rehabilitation also varies across cognitive domains, [31] highlighting the importance of offering personalised and tailored rehabilitation programs after stroke.

Our sensitivity analysis suggested that the change in cognitive function following haemorrhagic stroke, rather than ischaemic stroke, are greater, particularly for 3MS (global cognition) and HVLT-R (episodic memory) scores at the first cognitive assessment after stroke. These findings align with the involvement of small vessel pathology in haemorrhagic stroke which is shared with vascular cognitive impairment including cerebral amyloid angiopathy (CAA). CAA is a major cause of intracerebral haemorrhage in this age group, and inherently associated with cognitive decline. These findings may also be confounded by stroke severity, as intracerebral haemorrhage is generally more severe than acute ischemic stroke. Future studies should investigate specific features of stroke, such as type of stroke, size, location, and how these can impact cognitive trajectories.

Strengths of our study are the large prospective cohort of older adults who did not have any cardiovascular disease or major cognitive impairment at baseline. Adjudication of stroke and dementia were undertaken by a group of experts using associated medical records. The main limitation of this study was that we were unable to account for some key stroke features, such as location of stroke which might be predictive of dementia after stroke [7]. The short follow-up period following incident stroke (median 2.8 years) limited the number of dementia cases after incident stroke. A longer follow-up might also provide a better estimate of longer-term cognitive trajectories.

In conclusion, in this prospective cohort of initially healthy older individuals, stroke was associated with decreases in all four domains of cognitive function both at the first cognitive assessment following stroke and, in the years, following stroke though the magnitude of impact varied across the different cognitive domains. After incident stroke, participants experience steeper decline in global cognition, episodic memory and executive function and verbal fluency compared with stroke-free and pre-stroke trajectories. These findings highlight the need for comprehensive neuropsychology assessments for ongoing monitoring of cognition following incident stroke.

### Electronic supplementary material

Below is the link to the electronic supplementary material.

Supplementary Material 1

## Data Availability

The datasets generated during the current study are available by request to the corresponding author after approval from the ASPREE Pricncipal Investigators.
