# Supplementary material for: The impact of incident stroke on cognitive trajectories in later life
Source: Alzheimers Res Ther. 2024 May 18;16:111. doi: 10.1186/s13195-024-01479-8 (PMC11102228; doi:10.1186/s13195-024-01479-8)
Supplement: Supplementary file 1 — Supplementary Material 1 [file 13195_2024_1479_MOESM1_ESM.docx]

**Supplementary Tables**

| **ST1. Mean cognitive scores for participants in the study over follow-up, by stroke status (n=19,114)** | | | | | | | | |
| --- | --- | --- | --- | --- | --- | --- | --- | --- |
|  | **No stroke**  **n=18,299** | | | | **Stroke**  **n=815** | | | |
|  | **3MS** | **HVLT-R** | **SDMT** | **COWAT** | **3MS** | **HVLT-R** | **SDMT** | **COWAT** |
| **Year 1** | 94.2  (4.7) | 8.2  (3.0) | 37.0  (10.1) | 13.2  (4.8) | 93.5  (5.1) | 7.8  (3.3) | 33.8  (10.4) | 12.9  (5.1) |
|  |  |  |  |  |  |  |  |  |
| **Year 2*** | 84.4  (9.0) | 5.2  (3.7) | 27.8  (12.2) | 11.3  (4.9) | 82.5  (12.0) | 4.0  (2.8) | 25.0  (0) | 6.0  (1.4) |
|  |  |  |  |  |  |  |  |  |
| **Year 3** | 93.9  (5.3) | 8.2  (3.2) | 36.1  (10.1) | 13.4  (4.9) | 92.8  (6.7) | 7.6  (3.5) | 32.6  (10.4) | 12.7  (5.3) |
|  |  |  |  |  |  |  |  |  |
| **Year 4** | 94.1  (5.6) | 8.2  (3.2) | 36.1  (10.3) | 13.4  (4.8) | 92.3  (7.6) | 7.4  (3.7) | 30.8  (11.8) | 13.1  (5.3) |
|  |  |  |  |  |  |  |  |  |
| **Year 5** | 93.8  (6.1) | 8.2  (3.3) | 35.3  (10.1) | 13.6  (5.1) | 92.4  (8.2) | 7.5  (3.4) | 30.6  (10.4) | 13.0  (5.5) |
|  |  |  |  |  |  |  |  |  |
| **Year 6** | 94.0  (6.3) | 8.4  (3.2) | 35.1 (10.3) | 13.6  (5.1) | 92.5  (7.4) | 7.7  (3.5) | 30.8 (10.6) | 12.8  (4.9) |
|  |  |  |  |  |  |  |  |  |
| **Year 7** | 93.9  (6.6) | 8.3  (3.3) | 34.2  (10.2) | 13.5  (5.1) | 92.9  (6.5) | 7.8  (3.2) | 30.4  (10.6) | 11.7  (5.8) |
|  |  |  |  |  |  |  |  |  |
| **Year 8** | 93.7  (6.9) | 8.4  (3.3) | 33.4  (10.3) | 13.6  (5.2) | 92.1  (7.9) | 7.8  (3.5) | 29..3  (10.5) | 12.0  (5.2) |
|  |  |  |  |  |  |  |  |  |
| **Year 9** | 93.4  (7.0) | 8.3  (3.3) | 32.7  (10.5) | 13.6  (5.3) | 92.8  (6.7) | 7.7  (3.3) | 27.4  (9.8) | 11.9  (5.2) |
|  |  |  |  |  |  |  |  |  |
| **Year 10** | 93.3  (7.5) | 8.3  (3.5) | 31.9  (10.1) | 13.7  (5.4) | 92.9  (7.6) | 7.8  (3.9) | 29.3  (9.6) | 13.2  (5.2) |
|  |  |  |  |  |  |  |  |  |
| **Year 11** | 93.0  (8.9) | 8.3  (3.3) | 31.7  (10.6) | 13.6  (5.2) | 91.8  (7.5) | 8.7  (2.3) | 30.9  (9.9) | 12.7  (6.7) |
| **Note:**  * Year 2 cognitive assessments not included in the analysis  *Abbreviations:* n – sample size; 3MS – Modified Mini-Mental State exam; HVLT-R – Hopkins verbal learning test-revised; SDMT – symbol digit modalities test; COWAT – single letter controlled oral word association test. | | | | | | | | |

| **ST2. Stroke and change in cognitive performance over time (n=19,114) [complete model – including covariates]** | | | | | |
| --- | --- | --- | --- | --- | --- |
|  | | **3MS**  **(Global cognition)** | **HVLT-R**  **(Episodic memory)** | **SDMT**  **(Processing speed)** | **COWAT**  **(Verbal fluency)** |
| Overall cognitive change over time | | **-0.20**  **[-0.21, -0.18]** | **-0.04**  **[-0.04, -0.03]** | **-0.68**  **[-0.70, -0.67]** | **0.09**  **[0.08, 0.10]** |
| Acute cognitive change after stroke | | **-1.03**  **[-1.45, -0.60]** | **-0.47**  **[-0.70, -0.24]** | **-2.82**  **[-3.57, -2.08]** | **-0.67**  **[-1.04, -0.29]** |
| Cognitive change over time after stroke | | **-0.75**  **[-0.98, -0.53]** | **-0.12**  **[-0.70, -0.24]** | **-0.30**  **[-0.56, -0.05]** | **-0.17**  **[-0.28, -0.07]** |
| Age | | **-0.21**  **[-0.23, -0.20]** | **-0.16**  **[-0.16, -0.15]** | **-0.72**  **[-0.75, -0.70]** | **-0.06**  **[-0.07, -0.04]** |
| Sex | [Female] | **1.40**  **[1.27, 1.52]** | **1.09**  **[1.01, 1.17]** | **2.91**  **[2.65, 3.17]** | **1.40**  **[1.28, 1.52]** |
| Country and Ethnicity | [Aus/white] | **Ref** | **Ref** | **Ref** | **Ref** |
|  | [US/white] | **0.52**  **[0.27, 0.77]** | 0.09  [-0.06, 0.25] | **1.41**  **[0.89, 1.92]** | **-0.79**  **[-1.03, -0.54]** |
|  | [Afr/Amer] | **-2.68**  **[-2.98, -2.38]** | **-1.31**  **[-1.49, -1.12]** | **-5.88**  **[-6.49, -5.26]** | **-2.19**  **[-2.49, -1.90]** |
|  | [Other] | **-2.71**  **[-3.08, -2.33]** | **-0.29**  **[-0.53, -0.06]** | **-2.63**  **[-3.39, -1.87]** | **-1.51**  **[-1.87, -1.15]** |
| Education | [≥12yrs] | **1.82**  **[1.69, 1.94]** | **0.81**  **[0.73, 0.88]** | **4.40**  **[4.15, 4.66]** | **2.04**  **[1.92, 2.16]** |
| Smoking | [Current] | **Ref** | **Ref** | **Ref** | **Ref** |
|  | [Former] | **0.75**  **[0.43, 1.07]** | 0.16  [-0.04, 0.36] | **2.89**  **[2.22, 3.53]** | **0.52**  **[0.20, 0.83]** |
|  | [Never] | **0.85**  **[0.53, 1.17]** | **0.25**  **[0.05, 0.45]** | **3.08**  **[2.43, 3.73]** | **0.38**  **[0.07, 0.69]** |
| Alcohol | [Current] | **Ref** | **Ref** | **Ref** | **Ref** |
|  | [Former] | **-1.03**  **[-1.29, -0.78]** | **-0.49**  **[-0.65, -0.33]** | **-2.24**  **[-2.77, -1.71]** | **-0.42**  **[-0.67, -0.17]** |
|  | [Never] | **-0.74**  **[-0.90, -0.57]** | **-0.42**  **[-0.52, -0.31]** | **-1.36**  **[-1.70, -1.03]** | **-0.67**  **[-0.82, -0.44]** |
| Hypertension | [Yes] | **-0.28**  **[-0.42, -0.14]** | -0.08  [-0.17, 0.00] | **-0.73**  **[-1.01, -0.45]** | **-0.24**  **[-0.37, -0.11]** |
| Diabetes | [Yes] | **-0.65**  **[-0.84, -0.45]** | **-0.32**  **[-0.52, -0.31]** | **-1.35**  **[-1.75, -0.95]** | **-0.62**  **[-0.82, -0.44]** |
| Dyslipidemia | [Yes] | -0.07  [-0.20, 0.05] | -0.03  [-0.11, 0.05] | 0.26  [-0.00, 0.52] | 0.11  [-0.02, 0.23] |
| Depression symptoms* | [Yes] | **-0.71**  **[-0.91, -0.51]** | **-0.35**  **[-0.47, -0.22]** | **-1.59**  **[-2.00, -1.19]** | **-0.32**  **[-0.51, -0.12]** |
| **Note:** This table includes mixed linear regression results with participant-specific random intercept and slope  Bold represents s**ignificant** result *p<0.05*  *Abbreviations:* n – sample size; 3MS – Modified Mini-Mental State exam; HVLT-R – Hopkins verbal learning test-revised; SDMT – symbol digit modalities test; COWAT – single letter controlled oral word association test, Letter F; Aus, Australia; US, United States; Afr/Amer, African American; yrs – years. | | | | | |

| **ST3. Stroke and change in cognitive performance over time after excluding individuals who have died (n=16,086)** | | | | |
| --- | --- | --- | --- | --- |
|  | **3MS**  **(Global cognition)** | **HVLT-R**  **(Episodic memory)** | **SDMT**  **(Processing speed)** | **COWAT**  **(Verbal fluency)** |
| Cognitive change over time* | **-0.14**  **[-0.15, -0.12]** | **-0.03**  **[-0.03, -0.02]** | **-0.66**  **[-0.67, -0.64]** | **0.09**  **[0.08, 0.10]** |
| Acute cognitive change after stroke^#^ | **-0.88**  **[-1.33, -0.44]** | **-0.40**  **[-0.64, -0.15]** | **-2.59**  **[-3.39, -1.79]** | **-0.68**  **[-1.09, -0.28]** |
| Cognitive change over time after stroke^ | **-0.47**  **[-0.67, -0.28]** | **-0.12**  **[-0.16, -0.14]** | **-0.31**  **[-0.59, -0.03]** | **-0.16**  **[-0.27, -0.05]** |
| **Note:**  This table includes mixed linear regression results with participant-specific random intercept and slope adjusting for age, sex, country and ethnicity, education, smoking, alcohol, diabetes, depressive symptoms and including time (years) and a time-varying stroke variable (all participants were event-free at baseline), and a time variable representing the time (years) after a stroke  Bold represents s**ignificant** result *p<0.05*  * This effect size shows the annual change in cognitive scores on each of the four tests.  ^#^  This effect size shows the acute change in cognitive scores after an incident stroke (compared to pre-stroke).  ^ This effect size shows the annual change in cognitive scores on each of the four tests after a stroke  *Abbreviations:* n – sample size; 3MS – Modified Mini-Mental State exam; HVLT-R – Hopkins verbal learning test-revised; SDMT – symbol digit modalities test; COWAT – single letter controlled oral word association test. | | | | |

| **ST4. Stroke and change in cognitive performance over time after excluding individuals who had a dementia diagnosis (n=17,897)** | | | | |
| --- | --- | --- | --- | --- |
|  | **3MS**  **(Global cognition)** | **HVLT-R**  **(Episodic memory)** | **SDMT**  **(Processing speed)** | **COWAT**  **(Verbal fluency)** |
| Cognitive change over time* | **-0.04**  **[-0.05, -0.03]** | -0.00  [-0.01, 0.00] | **-0.63**  **[-0.64, -0.61]** | **0.11**  **[0.09, 0.11]** |
| Acute cognitive change after stroke^#^ | **-0.62**  **[-1.00, -0.23]** | **-0.36**  **[-0.59, -0.13]** | **-2.75**  **[-3.35, -1.99]** | **-0.50**  **[-0.89, -0.11]** |
| Cognitive change over time after stroke^ | **-0.47**  **[-0.64, -0.29]** | **-0.10**  **[-0.18, -0.01]** | **-0.25**  **[-0.49, 0.00]** | **-0.18**  **[-0.28, -0.07]** |
| **Note:**  This table includes mixed linear regression results with participant-specific random intercept and slope adjusting for age, sex, country and ethnicity, education, smoking, alcohol, diabetes, depressive symptoms and including time (years) and a time-varying stroke variable (all participants were event-free at baseline), and a time variable representing the time (years) after a stroke  Bold represents s**ignificant** result *p<0.05*  * This effect size shows the annual change in cognitive scores on each of the four tests.  ^#^  This effect size shows the acute change in cognitive scores after an incident stroke (compared to pre-stroke).  ^ This effect size shows the annual change in cognitive scores on each of the four tests after a stroke  *Abbreviations:* n – sample size; 3MS – Modified Mini-Mental State exam; HVLT-R – Hopkins verbal learning test-revised; SDMT – symbol digit modalities test; COWAT – single letter controlled oral word association test. | | | | |

| **ST5. Haemorrhagic stroke and change in cognitive performance over time (n=18,434 \| n=135 with stroke)^†^** | | | | |
| --- | --- | --- | --- | --- |
|  | **3MS**  **(Global cognition)** | **HVLT-R**  **(Episodic memory)** | **SDMT**  **(Processing speed)** | **COWAT**  **(Verbal fluency)** |
| Cognitive change over time* | **-0.20**  **[-0.21, -0.18]** | **-0.04**  **[-0.04, -0.03]** | **-0.68**  **[-0.70, -0.67]** | **0.09**  **[0.08, 0.10]** |
| Acute cognitive change after stroke^#^ | **-3.71**  **[-5.01, -2.41]** | **-1.21**  **[-1.93, -0.50]** | **-4.13**  **[-6.43, -1.82]** | 0.25  [-0.93, 1.43] |
| Cognitive change over time after stroke^ | **-1.00**  **[-1.69, -0.31]** | 0.13  [-0.13, 0.39] | -0.61  [-1.48, 0.25] | **-0.43**  **[-0.82, -0.05]** |
| **Note:**  This table includes mixed linear regression results with participant-specific random intercept and slope adjusting for age, sex, country and ethnicity, education, smoking, alcohol, diabetes, depressive symptoms and including time (years) and a time-varying stroke variable (all participants were event-free at baseline), and a time variable representing the time (years) after a stroke. Potential confounding factors not significant in the univariate analyses (hypertension, obesity, dyslipidaemia, and chronic kidney disease) were not included as covariates.  Bold represents **significant** result *p<0.05*  ^†^ From the total sample (n=19,114) deleted all ischemic stroke (n=596) and unknown/other stroke (n=84)  * This effect size shows the annual change in cognitive scores on each of the four tests.  ^#^  This effect size shows the acute change in cognitive scores after an incident stroke (compared to pre-stroke).  ^ This effect size shows the annual change in cognitive scores on each of the four tests after a stroke  *Abbreviations:* n – sample size; 3MS – Modified Mini-Mental State exam; HVLT-R – Hopkins verbal learning test-revised; SDMT – symbol digit modalities test; COWAT – single letter controlled oral word association test. | | | | |

| **ST6. Ischemic stroke and change in cognitive performance over time (n=18,895 \| n=596 with stroke)^†^** | | | | |
| --- | --- | --- | --- | --- |
|  | **3MS**  **(Global cognition)** | **HVLT-R**  **(Episodic memory)** | **SDMT**  **(Processing speed)** | **COWAT**  **(Verbal fluency)** |
| Cognitive change over time* | **-0.19**  **[-0.21, -0.18]** | **-0.04**  **[-0.04, -0.03]** | **-0.68**  **[-0.70, -0.67]** | **0.09**  **[0.08, 0.10]** |
| Acute cognitive change after stroke^#^ | **-0.69**  **[-1.16, -0.22]** | **-0.37**  **[-0.62, -0.12]** | **-2.68**  **[-3.49, -1.88]** | **-0.75**  **[-1.16, -0.34]** |
| Cognitive change over time after stroke^ | **-0.64**  **[-0.87, -0.42]** | **-0.13**  **[-0.22, -0.04]** | -0.22  [-0.48, 0.04] | **-0.14**  **[-0.25, -0.04]** |
| **Note:**  This table includes mixed linear regression results with participant-specific random intercept and slope adjusting for age, sex, country and ethnicity, education, smoking, alcohol, diabetes, depressive symptoms and including time (years) and a time-varying stroke variable (all participants were event-free at baseline), and a time variable representing the time (years) after a stroke. Potential confounding factors not significant in the univariate analyses (hypertension, obesity, dyslipidaemia, and chronic kidney disease) were not included as covariates.  Bold represents s**ignificant** result *p<0.05*  ^†^ From the total sample (n=19,114) deleted all haemorrhagic stroke (n=135) and unknown/other stroke (n=84)  * This effect size shows the annual change in cognitive scores on each of the four tests.  ^#^  This effect size shows the acute change in cognitive scores after an incident stroke (compared to pre-stroke).  ^ This effect size shows the annual change in cognitive scores on each of the four tests after a stroke  *Abbreviations:* n – sample size; 3MS – Modified Mini-Mental State exam; HVLT-R – Hopkins verbal learning test-revised; SDMT – symbol digit modalities test; COWAT – single letter controlled oral word association test. | | | | |

**Supplementary Figure**

| **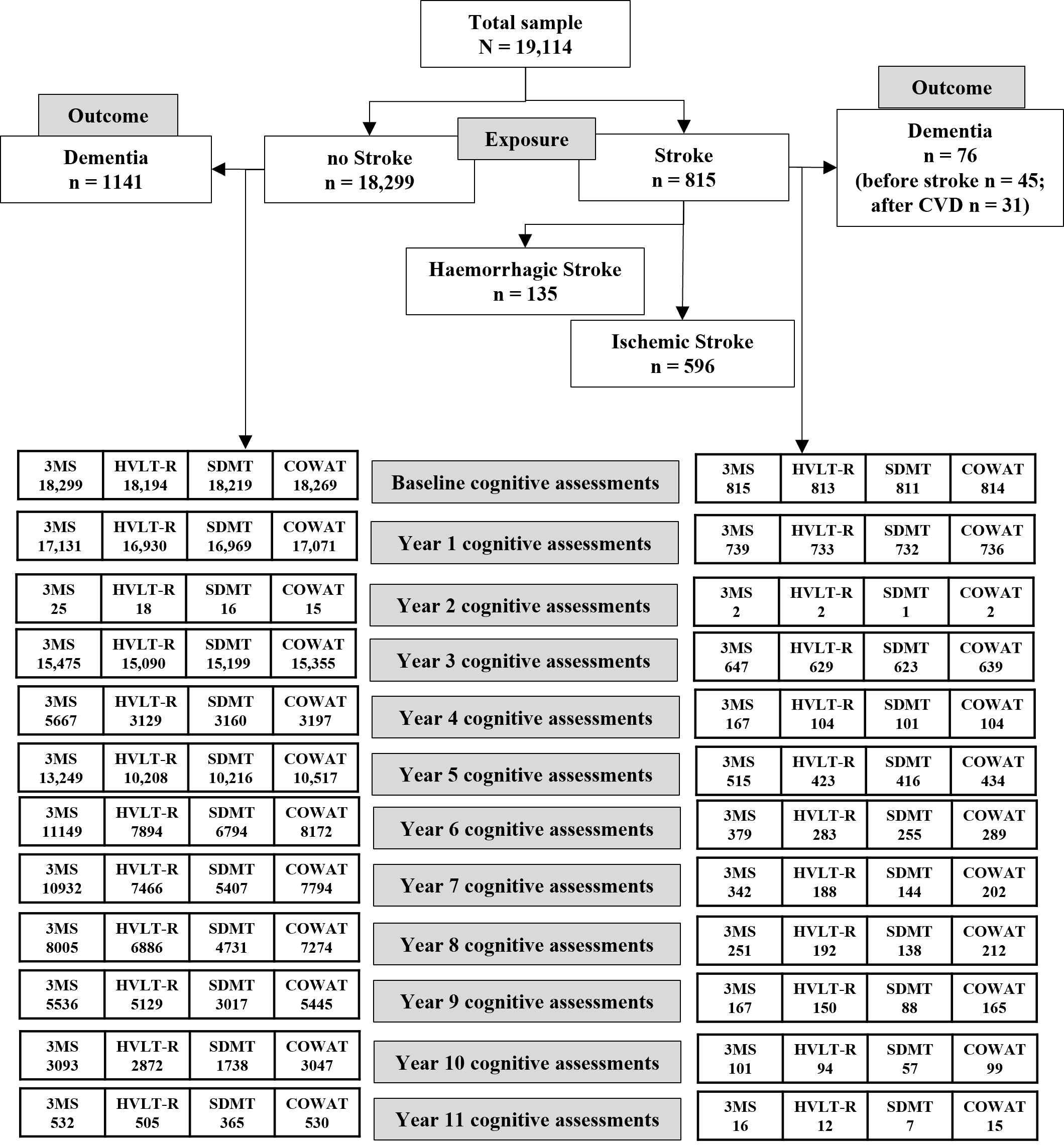** |
| --- |
| **SF1. Participant flow diagram** |
